# Evaluation of clinical trials for natural products used in diabetes: An evidence-based systemic literature review

**DOI:** 10.1097/MD.0000000000025641

**Published:** 2021-04-23

**Authors:** Rizwan Ahmad, Lina Hussain AlLehaibi, Hind Nasser AlSuwaidan, Ali Fuad Alghiryafi, Lyla Shafiq Almubarak, Khawlah Nezar AlKhalifah, Hawra Jassim AlMubarak, Majed Ali Alkhathami

**Affiliations:** a Natural Products and Alternative Medicines, College of Clinical Pharmacy, Imam Abdul Rahman Bin Faisal University, Kingdom of Saudi Arabia; b First Health Cluster in Eastern Province, Dammam Medical Complex, Dammam, Saudi Arabia; c College of Clinical Pharmacy, King Abdulaziz University, Jeddah, Saudi Arabia; d College of Clinical Pharmacy, Imam Abdulrahman Bin Faisal University, Dammam, Kingdom of Saudi Arabia.

**Keywords:** clinical trials, Delphi, diabetes, Jadad, natural products, systemic review

## Abstract

**Background relevance:**

A plethora of literature is available regarding the clinical trials for natural products however; no information is available for critical assessments of the quality of these clinical trials.

**Aim of study:**

This is a first time report to critically evaluate the efficacy, safety and large scale applications of up-to-date clinical trials for diabetes, based on the three scales of Jadad, Delphi, and Cochrane.

**Methodology:**

An in-depth and extensive literature review was performed using various databases, journals, and books. The keywords searched included, “clinical trials,” “clinical trial in diabetes,” “diabetes,” “natural products in diabetes,” “ethnopharmacological relevance of natural products in diabetes,” etc.

**Results:**

Based on eligibility criteria, 16 plants with 74 clinical trials were found and evaluated. Major drawbacks observed were; “non-randomization and blindness of the studies,” “non-blindness of patients/healthcare/outcome assessors,” “lack of patient compliance and co-intervention reports,” “missing information regarding drop-out/withdrawal procedures,” and “inappropriate baseline characteristics.” Principal component analysis and Pearson correlation revealed four components with %variability; PC1: 23.12, PC2: 15.83, PC3: 13.11, and PC4: 11.38 (*P* ≤ .000). According to descriptive statistics, “non-blinding of outcome assessors” was the major drawback (82%) whereas, “not mentioning the timing of outcome assessment” was observed lowest (6.8%). An in-house quality grading (scale 0–24) classified these clinical trials as; poor (67.6%), acceptable (19.9%), and good quality trials (13.5%).

**Conclusion:**

Proper measures in terms of more strict regulations with pharmacovigilance of plants are utmost needed in order to achieve quality compliance of clinical trials.

## 1 Introduction

“Diabetes Mellitus,” a term coined from Greek language where “Diabetes” stands for “a passer through” and “Mellitus” for “sweet.”^[1]^ Diabetes mellitus (DM) is a metabolic disorder which leads to chronic hyperglycemia, the pathogenesis for which may include defects in insulin secretion, action or both.^[2]^ Chronic autoimmune disease is considered a dominant cause behind insulin-dependent-diabetes (IDDM) which selectively destructs insulin secreting pancreatic β-cells and is treated by insulin. Non-insulin dependent diabetes mellitus (NIDDM) or type 2 diabetes is caused; due to insufficient insulin secretion via dysfunctional pancreatic β-cell, or insulin dysfunction due to decreased insulin sensitivity. First line treatment for NIDDM includes diet control and lifestyle modification; however, in case the diseases progresses, the use of oral hypoglycemic drugs is considered the next approach for treatment.^[3,4]^ According to International Diabetes Federation (IDF) report in 2019; the estimated population with diabetes was 463 million adults which has been projected to raise up to 578 M adults by 2030 and 700 M by 2045.^[5]^ This urges a proper treatment plan in order to lessen the prevalence of diabetes. The researchers are focusing more on natural products strategies in order to find an appropriate cure for diabetes. Several natural products have been successfully utilized to reduce the blood glucose level in the shape of pre-clinical and clinical studies.^[6]^ For instance, Magnesium (Mg) has been applied to recover Mg deficiencies and help relieve insulin resistance,^[7]^ cinnamon (known for insulin-like effect) has been reported to decrease blood sugar,^[8]^ and zinc has been studied to regulate insulin receptors and extend insulin action.^[9]^ Likewise, numerous plants have been observed with prominent folklore applications in various communities such as; bitter melon is considered a traditional plant to treat diabetes in Asia, South America, India, the Caribbean and East Africa,^[10]^ and fenugreek is used since long to cure diabetes in Mediterranean, Asian, North African, and European communities.^[11]^ Clinical research is a wide term that describe studies or trials conducted in human population^[12]^ with a vital role in developing new treatments and advancing medical knowledge. Clinical trials are classified on the basis of goal of study; to treat, prevent, or reduce incidence of a disease.^[13]^ With regard to natural products, the history of clinical trials dates back to 1990 and a huge amount of clinical trials has been published since then. Post-prandial blood sugar levels, fasting blood glucose, HbA1c, and insulin sensitivity are amongst the tangible, realistic, and varied examples of measurable metrics which reflects the substantial picture of “the effect of these natural products on diabetes patients.” Fenugreek, gymnema, aloe, neem, and various other natural products are the outcome of such clinical trial which were allocated in various part of the world, illustrating positive antidiabetic effects via different pathways.^[14–18]^ However, most of the natural products were unable to make access to the market due to various factors. The quality of clinical trials and its evaluation based on the pre-set standards is one of the important factors to declare the fate of these natural product. Herein, the authors took a challenge to scrutinize all the natural products, with an established ethnopharmacological background and reported clinical trial in diabetes, for evaluation of the quality of these clinical trials according to standard scales of Delphi, Jadad, and Cochrane based review scales.

## 2 Materials and methods

### 2.1 Databases and search strategy

*Electronic databases*; Scopus, PubMed, Google Scholar, Science Direct, and E-portal of Imam Abdulrahman Bin Faisal University library. *Journals*; Journal of medicinal plants research, Journal of ethnobiology and ethnomedicine, Asian journal of Plant sciences, Journal of diabetes and complications, BMC complementary and alternative medicines, Natural product research, Journal of Ethnopharmacology, Diabetes, Journal of diabetes, etc *Books*; Herbalism, phytochemistry and ethnopharmacology, indigenous drugs of India, etc.

The data selection/extraction/analysis and evaluation was performed by a group of graduate pharmacists, academicians, researchers, and physicians who were expert in the field of clinical trials and patient's treatment related to diabetes. The literature was double checked for any multiple publications/duplications, incomplete/ineligible study and scored according to the pre-defined scales (Jadad, Delphi, and Cochrane scale).

### 2.2 Keywords searched

Randomized clinical trial, clinical trials, diabetes mellitus, *Aloe vera*, *Panax quinquefolius*, American ginseng, *Vaccinium myrtillus*, Bilberry, *Cinnamomum cassia*, Cinnamon, *Trigonella foenum-graecum*, Fenugreek, *Allium sativum*, Garlic, *Gymnema sylvestre*, Gymnema, Karela, *Grifola frondosa*, Maitake, *Azadirachta indica*, Neem, *Opuntia fuliginosa*, Nopal, onion, *Allium cepa*, *Plantago ovata*, Psyllium, *Curcuma longa*, Turmeric, *Eleutherococcus senticosus*, *Acanthopanax senticosus*, ginseng, *Syzygium cumini,* Jambolana, Jambolan, Bitter melon.

### 2.3 Inclusion criteria

The inclusion criteria consisted of; “studies reported in English language only and reporting the natural products clinical trials for diabetes humans,” “natural products with established folklore uses and applied practically in diabetes trials,” “any clinical trial for diabetes using natural products irrespective of randomization, blinding, phase (I–V) applied, statistical model used, outcome and assessor blinding, and negative or positive outcomes,” “clinical trials reporting the use of natural products alongwith conventional medications.”

For ethnopharmacological relevance, a list of natural products was collected and final selection was based on the ethnopharmacological uses of these natural products. The relevant reports, based on community surveys, interviews, and collection of data from local inhabitants and healers in that particular community, were extracted.

### 2.4 Exclusion criteria

The criteria for literature exclusion was; “any study reporting diabetes clinical trials without application of natural products,” “clinical studies reported in diabetes using natural products with lack of any prior ethnopharmacological or folklore use,” “natural products with established ethnopharmacological uses in diabetes; however, yet to be evaluated in a proper clinical trial for diabetes,” “any preclinical, duplicated, and incomplete clinical trial,” “Phase-0 clinical trials,” and “clinical trials in diabetes using minerals, vitamins, or conventional drugs only.”

### 2.5 Review period

An extensive literature search strategy was applied which started in September 2018 and continued till February 2020. The literature was updated on regular basis, for any new information added to database, till final preparation of manuscript.

### 2.6 Ethical review

The ethical approval was not necessary as the study did not include any animal or human subjects.

### 2.7 Patient consent

The study did not involve any patients and no consent form was required.

### 2.8 Search result

**Figure 1:**
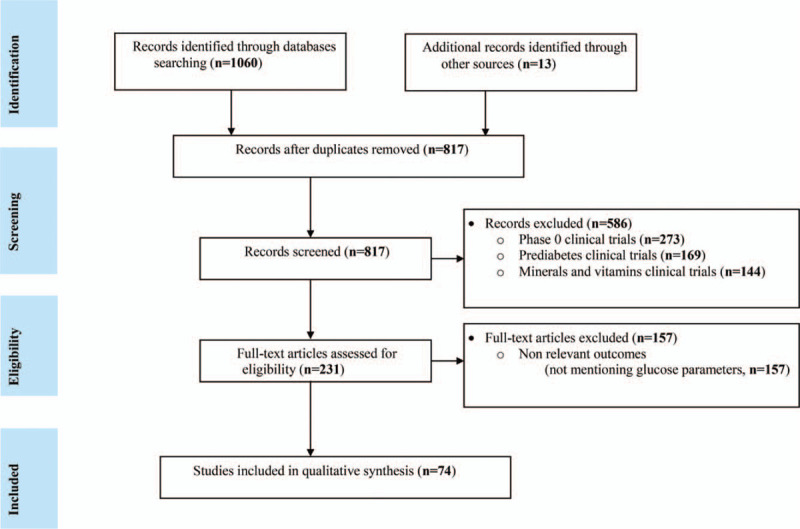
PRISMA flow-diagram for literature search and selection.

The literature search resulted a total of 1073 articles which were confined to 74 studies, following a proper scrutiny of the eligible articles as per pre-defined criteria (Fig. 1).

## 3 Literature search

The selected literature was downloaded, properly arranged, and studied in-depth for extraction of the relevant data. The bulk of this literature with relevant data is arranged in proper sections as mentioned below;

### 3.1 Ethnopharmacological relevance and evidence of the plants used in diabetes

Plants with ethnopharmacological relevance, part/s used for folklore purposes, and the community where the plants were applied for the treatment of various ailments have been reported in detail in Table 1.

**Table 1:** Ethnopharmacological relevance of the selected antidiabetic plants.

Herbs/plants	Botanical name	Synonyms	Part used	Ethnopharmacological relevance
Aloe	*Aloe vera*	*Aloe barbadensis/chinensis/ elongate/indica* Royle, Aloe	Leaves, dried sap (fluid), gel	Central and south America and Mexican communities, central Uganda^[46]^
American ginseng	*Panax quinquefolius*	*Panax quinquefolius*, *Panacis quinquefolius*, ginseng	Roots	different parts of the world^[47]^ and Quebec^[48]^
Bilberry	*Vaccinium myrtillus*	*Myrtillus niger and sylvaticus*, *Vaccinium oreophilum*	Fresh fruit	America and Europe,^[49]^ Canada^[48]^
Cinnamon	*Cinnamomum aromaticum; Cinnamomum cassia*	*Cinnamomum aromaticum/ longifolium/medium*, cinnamon	Bark, leaf	^[50,51]^
Fenugreek	*Trigonella foenum-graecum*	*Foenum-graecum officinale, Trigonella tibetana* (Alef.)	Seeds	Iran^[52–54]^
Garlic	*Allium sativum*	*Allium controversum* Schrad, *Allium longicuspis* Regel	Bulb, leaves	^[55–58]^
Gymnema	*Gymnema sylvestre*	gurmarbooti, gurmar, periploca	Dried roots and leaves	India and Africa,^[59]^ India, America^[47]^ and Canada^[48]^
Jambolan seeds	*Syzygium cumini; Syzygium cumini jambolana*	*Calyptranthes caryophyllifolia/ cumin/cuminodora/ jambolana*	Fruit, leaves, dried seed and bark	India,^[60,61]^ Khulna, Bangladesh^[62]^
Bitter melon	*Momordica charantia*	karela, bitter gourd	Fruit, leaf and whole plant	Dhaka, Bangladesh,^[63]^ Indian Jodhpur and Rajasthan communities^[64]^
Maitake	*Grifola frondosa*	Maitake mushroom	Fruits	Asia,^[65]^ China^[66]^
Neem	*Azadirachta indica*	Neem, neem tree or Indian lilac	Seeds/leaves/bark	India^[67–69]^
Nopal	*Opuntia fuliginosa/ streptacantha*	Prickly pear cactus, nopal	Flowers, fruits	Morocco,^[70]^ Latinos and Hispanics^[71]^
Onion	*Allium cepa*	*Allium angolense/aobanum/cepaeum*	Leaves, bulb, oil and seeds	Tamilnadu, India,^[69]^ Palestine^[72]^
Psyllium	*Plantago ovata*	plantago seeds, psyllium husk	Seeds, husk	Mexico^[73,74]^
Siberian Ginseng	*Eleutherococcus senticosus; Acanthopanax senticosus*	*Acanthopanax asperatus, Eleutherococcus asperatus*	Bark, roots	^[75,76]^
Turmeric	*Curcuma longa/ domestica; Curcuma aromatica*	*Amomum curcuma* Jacq*, Curcuma domestica* Valeton, curcumin	Whole plant, fresh rhizome	^[67]^, India^[77]^

### 3.2 Clinical trials of plants used for diabetes

This section describes in detail, the clinical trials reported for natural products in diabetes. A comprehensive information regarding the part of the plant used in the study, mechanisms of action reported, and final results observed are given in Table 2.

**Table 2:** Details regarding plant, its part used, mechanism reported and results observed in clinical trials during diabetes.

Plant	Clinical trail	Part used	Sample size (n)	Intervention method in intervened groups	Period of treatment	Mechanism reported	Results
Aloe vera	A1	Juice from gel	72	1 tablespoonful aloe juice BID	42 days	N/A	↓Blood sugar and triglyceride levels^[78]^
	A2	Juice from gel	72	1 tablespoonful aloe juice BID + 2 glibenclamide (5 mg) tablets	42 days	N/A	↓ Glucose and ↓ triglyceride^[79]^
	A3	Leaves extracted gel powder	67	Aloe capsules (300 mg BID)	2 months	↓Insulin resistance	↓HbA1c, LDL, and total cholesterol^[80]^
	A4	Extract as tablet	44	Aloe extract tablets (1000 mg OD)	2 months	N/A	No reduction in fasting blood sugar, HbA1c, total cholesterol, triglycerides, HDL/LDL^[81]^
	A5	Powder of the leaves extracted gel	90	First intervention: Group 1: no treatment. Group 2: aloe powder (100 mg) Group 3: aloe powder (200 mg)	3 months	↑Effectiveness of insulin	↓Fasting and post prandial blood glucose and lipid profile^[82]^
				Second intervention: nutrition to group 2 and 3.	3 months		
American ginseng	B1	Opaque gelatin capsule	39	Konjac-glucomannan blend fiber (6g/day) + ginseng (3g/day)	12 weeks with 4 weeks washout period (Crossover)	↑insulin secretion	↓ HbA1c and lipid panel^[83]^
	B2	Ginseng root extract	74	Capsules (total 3g/day)	12 weeks	N/A	Safe in T2DM patient with CVS risk (Mucalo et al., 2014)
	B3	Gelatin capsules	19	Ginseng capsules (3g) + oral glucose challenge (25g) in each visit	4 visits (1 week interval between each visit)	↓Digestion and ↑insulin secretion	Change in glycaemia^[84]^
	B4	Root of American ginseng	10	Either placebo or ginseng 3, 6, or 9 g randomly/each visit	16 visits with a 3 days interval	↓Digestion and ↑insulin secretion	No effect on post prandial glycaemia^[85]^
	B5	Dried whole root extract	24	Ginseng capsules (3g/day)	8 weeks with 4 weeks washout period	↑Insulin secretion	↓HbA1c, fasting blood glucose^[86]^
Bilberry	C1	Fruit extract	8	0·47 g of Mirtoselect (equal to 50 g of fresh bilberries)	Single dose with 2 weeks washout period	↓Carbohydrate digestion or absorption	↓Postprandial glycaemia and insulin^[87]^
Cinnamon	D1	Capsule	60	Cinnamon capsules (500 mg BID)	3 months	NA	No change in glucose and lipid profile^[88]^
	D2	Aqueous extract as capsule	60	1, 3, 6 g cinnamon daily	40 days	↑Stimulation of insulin	↓Serum glucose and lipid profile^[89]^
	D3	Whole bark extract as capsule	25	Cinnamon 1500 mg/day	6–7 weeks	↑Insulin sensitivity	No improvement in glucose^[90]^
	D4	Capsule	109	Cinnamon capsule (1g/day)	90 days	N/A	↓HbA1c^[91]^
	D5	Aqueous cinnamon extract	79	Capsule (112 mg of aqueous cinnamon extract TID)	4 months	N/A	↓Fasting glucose^[92]^
	D6	Capsule	14	Giving cinnamon capsule 1.5g/day	30 days	NA	↓Glucose, triglycerides and cholesterol^[93]^
	D7	Bark extract as tablet	66	Placebo/cinnamon extract at 120 or 360 g/day.	3 months	NA	↓Fasting blood glucose and HbA1c^[94]^
	D8	Capsule	72	Cinnamon (1 g/day)	90 days	↑Insulin stimulated tyrosine phosphorylation	No significant differences in glucose profile or number of hypoglycemic episodes^[95]^
	D9	Bark extract as capsule	44	Cinnamon supplement (3g/day).	8 weeks	↑Insulin stimulated tyrosine phosphorylation	No significant difference in glycemic indicators between arms of the study^[96]^
	D10	Bark powder as capsule	58	Cinnamon (2 g/day)	12 weeks	↑Glucose transporter (GLUT4) and receptor proteins	↓HbA1c and blood pressure^[97]^
Fenugreek	E1	Seed extract (Fenfuro-TM)	174	Fenfuro capsule (500 mg BID)	90 days	NA	↓Post-prandial blood glucose and FBG^[98]^
	E2	Seeds soaked in hot water	60	Fenugreek seeds soaked in hot water (10g/day)	6 months	↑ Insulin release	↓FBG levels and HbA1c^[99]^
	E3	Dried ripe seed capsule	69	Fenugreek saponins 6 capsules (TFGs) TID (0.35g/cap)	12 weeks	NA	↓Glycemia and CSQS in the treated group^[100]^
	E4	Seed powder	24	Powdered fenugreek seeds (10 g/day) yogurt or with hot water.	8 weeks	↑Insulin	↓FBG^[101]^
	E5	Hydro-alcoholic seeds extract	25	Hydro-alcoholic extract (1g/ day)	2 months	↑Insulin release	↓HbA1c and insulin resistance^[102]^
	E6	Seed powder	80	Fenugreek powder (25g/day)	2 months	↑ Insulin release	↓FBS and HbA1c^[103]^
	E7	Seed powder	30	Two sachet of Polyherbal formulation (PHF) containing fenugreek 2.5 g	40 days	↑Insulin	↓FBG and HbA1c^[104]^
Garlic	F1	Aged garlic extract (Kyolic)	26	1200 mg of Aged garlic extract daily	4 weeks with 4 weeks washout	N/A	No extra benefit for adding aged garlic^[105]^
	F2	Aqueous extract capsule	32	Capsules (combination of 200 mg turmeric and 200 mg garlic). Three groups; group 1 (1.2g), group 2 (1.6g) and group 3 (2.4g) daily	12 weeks	↑ Insulin secretion	↓Glucose profile^[106]^
	F3	Garlic powder tablets (Allicor)	60	300 mg Allicor/day	4 weeks	↑Insulin secretion	↓Blood glucose^[107]^
	F4	Bulb extract (Lasuna) capsule	60	Garlic capsules (250 mg BID) added to standard therapy	12 weeks	↑Insulin secretion and sensitivity	↓Glycemic level^[108]^
	F5	N/A	96	Capsules (50 mg/day) added to standard medication	12 weeks	N/A	↓Fasting blood glucose^[109]^
	F6	Garlic (KWAI) tablet	60	Tablets (300 mg TID)	24 weeks	↑ Insulin secretion	↓Fasting blood sugar^[110]^
	F7	Garlic tablet	210	5 groups received garlic (300, 600, 900, 1200, and 1500 mg/day), one took metformin and one was placebo.	24 weeks	N/A	↓FBS and HbA1c^[111]^
	F8	Kyolic aged garlic extract	48	Extract (3g/day)	3 months	↑ Insulin secretion	No change in blood glucose^[17]^
	F9	Bulb extract (Lasuna) capsules	60	Capsules (250 mg BID) + metformin.	12 weeks	Sulfur containing metabolites i.e. allicin and its derivatives	Reduction in fasting blood glucose when used with metformin (250 mg)^[112]^
Gymnema	G1	Water-soluble leaves extract	60	2 capsules daily (200 mg/cap)	2–30 months	↑Endogenous insulin	↓Insulin requirements^[14]^
	G2	Water-soluble leaves extract	47	Capsule (400 mg/day)	18–20 months	Beta cells regeneration	Reduced glucose and glycosylated Hgb^[15]^
	G3	Beta Fast GXR (leaves extract)	100	Tablets (400 mg BID)	90 days	↑Insulin levels due to regeneration of the pancreatic beta cells	↓Postprandial plasma glucose, HbA1c and pre-prandial plasma glucose concentrations^[113]^
	G4	Om Santal Adivasi (OSA)	11	Capsules (1 g/day)	6 days	↑Insulin secretion	↓Fasting glucose and ↑serum insulin^[114]^
	G5	Leaves powder	20	Powder (6 g/day)	One month	N/A	↓Blood glucose and postprandial blood glucose levels^[115]^
Jambolan	H1	Dried leaves tea	27	Group 1: *Syzygium cumini* leaves tea (2g/ day) + placebo tablets, Group 2: placebo tea + glyburide tablets (5 mg BID), Group3: placebo tea & tablet	28 days	N/A	No hypoglycemic effect observed^[116]^
	H2	Dried leaves tea	27	*Syzygium cumini* leaves tea (2g/day) + placebo tablets, placebo tea + glyburide tablets (5 mg BID), placebo tea + placebo tablet	28 days	N/A	↓Hyperglycemic effect^[117]^
Bitter Melon	I1	Powdered whole karela fruit	8	Powder 50 mg/kg BID	7 days	N/A	Enhanced glucose tolerance^[118]^
	I2	Dried powder of fruit pulp in capsule	129	Karela 0.5 g/day, 1 g/day, 2 g/day or metformin 1 g/day.	4 weeks	N/A	↓Fructosamine however, the hypoglycemic effect was less than metformin^[119]^
	I3	Fresh whole fruit in tablet	50	Tablets (6 g/day) + standard medication	4 weeks	N/A	No significant changes observed^[120]^
	I4	Methanolic fruit soft extract	15	Extract (200 mg BID) + half dose metformin/glibenclamide	7 days	N/A	↑Hypoglycemic action^[121]^
	I5	Dried fruit pulp capsule	95	Karela (2/4 g/day) or glibenclamide (5 mg/day)	10 weeks	N/A	↓HbA1c and plasma glucose^[122]^
	I6	Extract of fruits/tissue cultures	9	Vegetable insulin doses (10/20/30 IU)	One day (single dose)	N/A	↓Blood glucose^[123]^
	I7	Dried fruit pulp capsule	24	Capsule (2000 mg/day)	3 months	↑Insulin secretion	↓HA1c and insulin AUC^[124]^
	I8	Charantia Ampalaya capsules	40	2 capsule TID	3 months	N/A	No significant effect observed^[125]^
	I9	Fresh unripe fruit juice	50	Rosiglitazone (4 mg/day) or bitter melon juice (55 mL/24 h)	6 months	N/A	No change in serum glucose^[126]^
Maitake	J1	Fruit bodies in caplets	Two	First participant; MMP caplet (500 mg TID) reduced to 2caplets/ day	5 months	↑Effect on insulin receptors	↑Glycemic control^[127]^
				Second participant; MMP caplet (500 mg TID)	3 months		
Neem	K1	Powder leave aqueous extract	400	Neem extracts (5 mL/day)	2 months	↓Carbohydrate absorption from gut	↓Fasting blood sugar level^[18]^
	K2	Powder leave aqueous extract	90	Capsules (2 g/day) of *tulsi* leaf powder, *neem* leaf powder or mixture of both	3 months	N/A	↓Diabetic symptoms^[128]^
	K3	Seeds powder	55	*Moringa oleifera* (8g) or neem (6g) per day	40 days	N/A	↓Fasting and postprandial blood glucose^[129]^
Nopal	L1	N/A	Study 1: 7	Meal containing 50 g carbohydrates from glucose or dehydrated nopal	1 visit/each meal (1 week washout period	N/A	↓Plasma glucose-dependent insulinotropic peptide peaks and serum insulin^[130]^
			Study 2: 14	High-carbohydrate breakfast or high-soy-protein breakfast, with/without 300 g of nopal	4 visits (1 week washout period between meals)		
	L2	Fresh and tender stems	32	Group 1: broiled nopal stems (500 g), group 2: only water (400 mL), Group 3: nopal, water and broiled squash	Single dose, 1 week washout between each intervention.	↑Insulin sensitivity is suggested	↓Glucose and ↑hypoglycemia^[131]^
	L3	Stems	28	500 g of nopal	Single dose	N/A	↓Serum glucose and insulin^[132]^
	L4	Dehydrated extract, capsulated	6	30 capsules of dehydrated nopal extract (10.1 g)	Single dose	N/A	No hypoglycemic effects observed^[132]^
	L5	Heated blended crude stem	8	5 interventions; 4 for nopal stems (entire broiled, blended broiled, blended crude, and heated blended) and 1 water as placebo.	5 separate interventions (72 hs between them)	N/A	↓Serum glucose^[132]^
	L6	Dried, capsulated	10	30 nopal capsules	Single dose	N/A	No hypoglycemic effect observed^[132]^
			14	10 nopal capsules TID.	1 week		
Onion	M1	Fresh onion cut into slices	84	Crude fresh slices (100 g), standard diabetic treatment, or 15 ml of water.	Single dose	Improved and regenerated cells	↓Blood glucose and FBG^[133]^
Psyllium	N1	Psyllium pre-mixed in cookies	77	Cookies containing either flaxseed/psyllium/placebo (10 g/day)	12 weeks	N/A	↓FBG and HbA1c^[134]^
	N2	Psyllium pre-mixed in cookies	51	cookies containing either flaxseed/ psyllium/placebo (10g per day)	12 weeks	N/A	↓FBG and HbA1c^[135]^
	N3	Fiber psyllium (Metamucil)	37	Psyllium (3.4 g BID), psyllium (6.8 g BID) or placebo.	12 weeks	↓Carbohydrate absorption	↓FBG and HbA1c^[136]^
	N4	Soluble fiber	40	Soluble fiber (10.5 g) daily	8 weeks	↓ CHO absorption/digestion	↓Glucose level^[137]^
	N5	Psyllium fiber	18	6.8 g psyllium twice in the first visit and placebo in the crossover visits.	One day of treatment for each group (crossover)	↓Access of glucose to the gut	↓PBG and insulin concentrations^[138]^
Siberian ginseng	O1	Purified solution of extract	75	Extract of Siberian ginseng (480 mg/day), American ginseng (480 mg/day), or placebo	3 months	↑Glucose induced insulin secretion	↓Fasting and post prandial blood sugar^[139]^
Turmeric	P1	(Sina Curcumin)	70	Curcumin (80 mg/day)	3 months	N/A	↓HbA1c, FBG, TG, and BMI^[140]^
	P2	Capsule	100	Curcuminoids capsule (150 mg BID)	3 months	↓Serum A-FABP levels	↓ Blood glucose with anti-diabetic effects^[141]^
	P3	Rhizomes	60	2 g turmeric + standard metformin therapy.	4 weeks	↑Beta cell stimulation	↓Fasting plasma glucose^[16]^
	P4	Extracts isolated from rhizome	100	Curcuminoids (300 mg/day)	3 months	↓BG and ↑insulin resistance	↓Fasting blood glucose^[142]^

### 3.3 Evaluation of clinical trials based on various scales

Jadad scale, is a tool used to assess the quality of clinical trials based on the three key features of randomization, masking, and accountability of all patients including the withdrawals. The Jadad system evaluates and scores a study on a scale of 0 to 5, based on the pre-defined factors as mentioned in Jadad scale (Table 3). Delphi uses numerous items to measure the quality of a clinical trial. Though a deficiency of a numerical scale for calculation of final score do exist in this system, yet it is widely implemented scale in most of the studies. Delphi uses nine designated questions in order to count the number of positive responses (Table 3). Cochrane back review group developed a scale which is considered the most comprehensive and uncritical among all the available scales. It is considered a quality rating standard scale, followed in most of the review studies for evaluation of the clinical trials. This system also has a lack of numerical scale needed to finalize a value for a clinical trial. As evident from Table 3, few of the points in the three mentioned scales are overlapping. In addition, each scale do possess positive and negative aspects hence, it becomes difficult to decide a best-fit scale for evaluation. To overcome the loopholes present in these scale the authors utilized a novel approach i.e. to apply the three scales together for evaluation of the quality of each clinical trial on individual basis. To maintain uniformity and ease of application in calculating the individual and final scores for each clinical trial, herein we calculated the responses from these two scales in terms of numerical values 0 to 9 (Delphi system) and 0 to 10 (Cochrane based review scale) which sum up to a final value of 0 to 24 points.^[19]^ The scales are applied to evaluate each individual trial and the drawbacks observed per each scale are reported given in Table 4.

**Table 3:** The scales used for evaluation of clinical trials.

Cochrane back review group list	Final Delphi list	Jadad score calculation
Was the method of randomization adequate?	1. Treatment allocation(a) Was a method of randomization performed?(b) Was the treatment allocation concealed?	Was the study described as randomized (this includes words such as randomly, random, and randomization)?
Was the treatment allocation concealed?		Was the method used to generate the sequence of randomization described and appropriate (table of random numbers, computer generated, etc.)?
Were the groups similar at baseline regarding the most important prognostic indicators?	2. Were the groups similar at baseline regarding the most important prognostic indicators?	Was the study described as double blind?
Was the patient blinded to the intervention?	3. Were the eligibility criteria specified?	Was the method of double blinding described and appropriate (identical placebo, active placebo, dummy, etc)?
Was the care provider blinded to the intervention?	4. Was the outcome assessor blinded?	Was there a description of withdrawals and dropouts?
Was the outcome assessor blinded to the intervention?	5. Was the care provider blinded?	Deduct one point if the method used to generate the sequence of randomization was described and it was inappropriate (patients were allocated alternately, or according to date of birth, hospital number, etc).
Were co-interventions avoided or similar?	6. Was the patient blinded?	Deduct one point if the study was described as double blind but the method of blinding was inappropriate (e.g., comparison of tablet vs injection with no double dummy).
Was the compliance acceptable in all groups?	7. Were point estimates and measures of variability presented for the primary outcome measures?	
Was the drop-out rate described and acceptable?	8. Did the analysis include an intention-to-treat analysis?	
Was the timing of the outcome assessment in all groups similar?		

**Table 4:** limitations, individual and total score calculated for each clinical trial based on Jadad, Delphi and Cochrane scales.

		Jadad deficiencies						Delphi deficiencies										Cochrane deficiencies											
Plant	Study #	a	b	c	d	e	Jadad Score^[5]^	a	b	c	d	e	f	g	h	i	Delphi score^[9]^	a	b	c	d	e	f	g	h	i	j	Cochrane score^[10]^	Total score^[24]^
Aloe vera	A1	X	X	X	X		1	X	X			X	X				3	X	X			X	X	X	X			1	5
	A2	X	X	X	X		1	X	X			X	X				3	X	X			X	X	X	X			1	5
	A3						5					X	X			X	6					X	X					8	19
	A4						5			X			X			X	5			X		X			X			6	16
	A5	X	X	X	X	X	0	X	X	X	X	X	X	X			1	X	X	X	X	X	X	X	X	X		0	1
American ginseng	B1			X	X		3						X	X		X	6				X	X		X				7	16
	B2						5					X	X			X	6					X	X					8	19
	B3		X	X	X		1		X		X	X	X				4	X	X			X	X		X			4	9
	B4			X	X		3		X	X	X	X	X				3		X	X		X	X		X			3	9
	B5						5						X				8					X		X				7	20
Bilberry	C1		X			X	2									X	7	X							X	X		5	14
Cinnamon	D1		X			X	2										9	X								X		7	18
	D2		X	X	X	X	0		X			X	X			X	4	X	X			X	X	X		X		1	5
	D3	X	X			X	2	X				X				X	5	X					X			X		6	13
	D4			X	X		3		X				X	X			6		X		X	X						7	16
	D5		X				3					X				X	6	X					X					7	16
	D6		X	X	X	X	0		X			X	X			X	5	X	X			X	X	X	X	X		1	6
	D7		X		X		1					X				X	6	X					X	X				4	11
	D8		X			X	2					X					7	X					X			X		5	14
	D9		X				3									X	8	X										9	20
	D10						5										9											10	24
Fenugreek	E1					X	4					X				X	5						X			X		7	16
	E2			X	X	X	2						X	X			6				X	X		X	X	X		3	11
	E3						5					X					7						X					8	20
	E4	X	X	X	X		1	X	X	X	X	X	X	X			-1	X	X	X	X	X	X	X	X			-3	-3
	E5		X			X	2		X		X	X					4	X	X				X		X	X		2	8
	E6		X	X	X		1		X	X		X	X	X			3	X	X	X	X	X	X					3	7
	E7	X	X	X	X		1	X	X			X	X	X		X	3	X	X		X	X	X		X			3	7
Garlic	F1		X		X		1					X				X	6	X					X					6	13
	F2		X		X		1					X					7	X					X					6	14
	F3		X		X		1										9	X							X			6	16
	F4	X	X	X	X	X	0	X	X			X	X	X		X	3	X	X		X	X	X			X	X	3	6
	F5		X	X	X	X	0		X			X	X	X	X	X	3	X	X		X	X	X	X	X	X		-1	2
	F6	X	X	X	X		1	X	X			X	X			X	3	X	X			X	X					5	9
	F7		X	X	X		1		X				X	X		X	4	X	X			X	X					4	9
	F8	X	X	X	X	X	0	X	X			X	X	X		X	3	X	X		X	X	X	X	X	X		0	3
	F9		X	X	X	X	0		X			X	X	X		X	4	X	X		X	X	X		X	X		1	5
Gymnema	G1	X	X	X	X		1	X	X			X	X	X			4	X	X		X	X	X					5	10
	G2	X	X	X	X	X	0	X	X			X	X	X		X	3	X	X		X	X	X			X		4	7
	G3	X	X	X	X		1	X	X			X	X	X		X	3	X	X		X	X	X					5	9
	G4	X	X	X	X	X	0	X	X	X		X	X	X		X	2	X	X	X	X	X	X			X		3	5
	G5	X	X	X	X	X	0	X	X			X	X	X		X	3	X	X		X	X	X			X		4	7
Jambolan	H1		X			X	2					X					8	X				X	X		X	X		5	15
	H2		X			X	2			X	X	X			X	X	3			X			X		X	X	X	2	7
Bitter Melon	I1		X	X	X	X	0		X	X		X	X	X			0	X	X	X	X	X	X					-1	-1
	I2						5			X		X					6			X			X					7	18
	I3		X	X	X		1		X				X			X	5	X	X		X			X				5	11
	I4	X	X	X	X	X	0	X	X	X		X	X	X		X	-4	X	X	X	X	X	X	X	X	X		-8	-12
	I5		X		X		1					X				X	6	X					X					6	13
	I6	X	X	X	X	X	0	X	X	X		X	X	X		X	-3	X	X	X	X	X	X			X		-2	-5
	I7						5					X					7						X					8	20
	I8						5										9											10	24
	I9		X	X	X	X	0		X			X	X	X		X	-1	X	X		X	X	X		X	X		-3	-4
Maitake	J1	X	X	X	X	X	0	X	X	X	X	X	X	X		X	1	X	X	X	X	X	X			X		3	4
Neem	K1		X	X	X		1		X	X		X	X	X		X	2	X	X	X	X	X	X					2	5
	K2	X	X	X	X	X	0	X	X			X	X	X		X	3	X	X		X	X	X			X		4	7
	K3	X	X	X	X	X	0	X	X			X	X	X		X	3	X	X		X	X	X			X		4	7
Nopal	L1	X	X	X	X		1	X	X			X	X	X		X	0	X	X		X	X	X		X			1	2
	L2		X	X	X	X	0		X		X	X	X	X		X	-2	X	X		X	X	X		X	X		-4	-6
	L3	X	X			X	2	X			X	X				X	3	X					X		X	X		3	8
	L4	X	X			X	2	X		X	X	X				X	0	X		X			X		X	X		1	3
	L5	X	X			X	2	X				X				X	3	X					X		X	X		3	8
	L6	X	X			X	2	X		X	X	X				X	0	X		X			X	X	X	X		-1	1
Onion	M1	X	X	X	X	X	0	X	X	X		X	X	X		X	0	X	X	X	X	X	X		X	X		1	1
Psyllium	N1			X	X		3		X			X	X			X	5		X			X	X	X				6	14
	N2			X	X		3		X	X		X	X			X	3		X	X		X	X					5	11
	N3		X		X		1			X					X	X	5	X		X					X		X	4	10
	N4		X	X	X		1		X	X		X	X	X		X	2	X	X	X	X	X	X	X	X			1	4
	N5		X	X	X	X	0		X	X		X	X	X		X	2	X	X	X	X	X	X			X		3	5
Siberian ginseng	O1		X		X	X	0					X	X				7	X				X	X		X	X		4	11
Turmeric	P1				X		3					X				X	7						X		X			8	18
	P2		X	X	X	X	0		X			X	X	X		X	2		X		X	X	X		X	X	X	1	3
	P3		X	X	X	X	0		X			X	X	X		X	2		X		X	X	X	X		X	X	1	3
	P4						5					X				X	7						X		X			8	20

### 3.4 Statistical analysis

**Figure 2:**
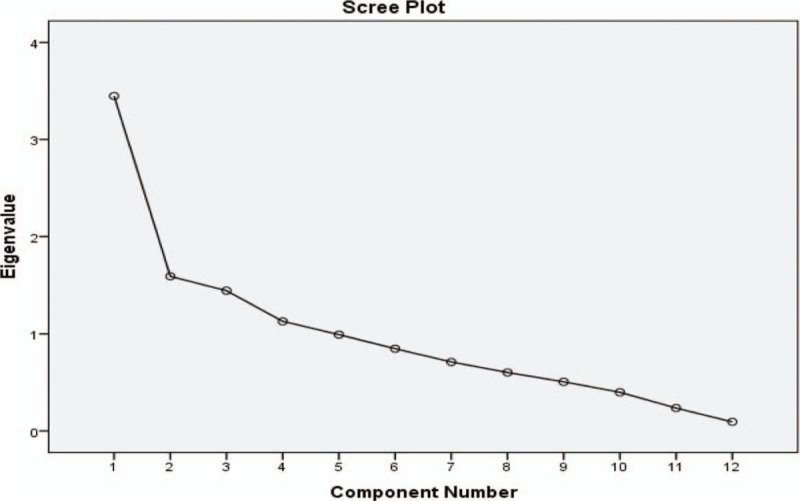
Scree plant, representing the number of possible components for factors loading.

**Figure 3:**
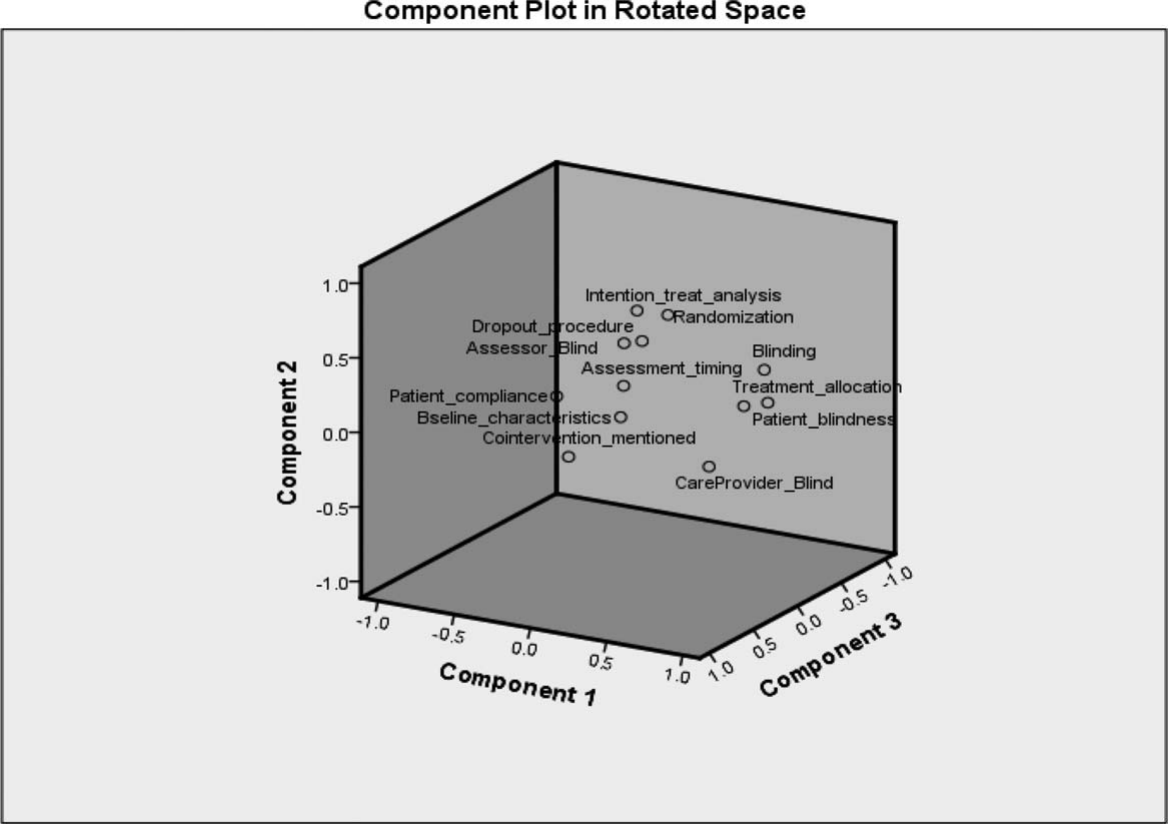
Scree plot and component loadings for factors.

For principal component analysis (PCA), four components (scree plot Fig. 2) were observed with %variability (individual and total); PC1: 23.12 (23.12), PC2: 15.83 (38.96), PC3: 13.11 (52.07), and PC4: 11.38 (63.45). A total of 63.45% variability with Kaiser–Meyer–Olkin Measure of Sampling Adequacy (0.659) and Bartlett's test chi square value of 289.58 (*P* ≤ .000) was observed (Table 5). Around 23.12% variability was observed for the drawbacks; “non-randomized and non-blinded studies,” “lack of patient and care provider blinding,” “concealed treatment allocation,” and “lack of proper drop-out procedures mentioned in the studies.” Whereas 15% variation was due to; “lack of/no compliance report for patients” and “no information about co-interventions applied in the study.” Furthermore, no information about the “intention-to-treat-analysis” and “timing of outcome assessment,” was loaded in PC3 with individual variation of 13.11%. Finally, “non-blindness of the outcome assessor” contributed 11.38% to the total variance (63.455%). A three-dimensional representation of the factors loaded in components is shown in Figure 3.

**Table 5:** Principal components loading (PCA) for factors analyzed in clinical studies and KMO and Bartlett's test.

Factors	PC1	PC2	PC3	PC4
(A) Clinical trial was randomized or not?	0.610	0.141	0.140	−0.463
(B) Clinical trial was blinded or non-blinded?	0.665	−0.556	0.010	−0.032
(C) Treatment allocation was concealed or not?	0.806	−0.141	−0.213	0.123
(D) The outcome assessor was blinded or not?	0.270	0.399	−0.157	−0.610
(E) Patient was blinded in the study or not?	0.866	−0.286	−0.217	0.125
(F) The care provider was blinded or not?	0.634	0.186	−0.543	0.281
(G) The intention to treat analysis, was mentioned in clinical trial?	0.416	−0.066	0.602	−0.197
(H) Proper drop out procedure was mentioned?	0.640	0.249	0.320	−0.098
(I) Was the patient compliance for the clinical trial reported?	0.285	0.624	0.328	0.206
(J) Was the timing of outcome assessment mentioned?	0.111	−0.267	0.628	0.352
(K) Was the baseline characteristics for the group mentioned?	0.233	0.121	0.203	0.299
(L) Was any co-interventions mentioned?	0.168	0.654	−0.068	0.356
Variability %	23.12	15.83	13.11	11.38
Cumulative %	23.12	38.96	52.07	63.455

KMO and Bartlett's test		
Kaiser–Meyer–Olkin measure of sampling adequacy	0.659	
Bartlett's test of sphericity	Approx. Chi-square	289.58
	degree of freedom	66
	Significance	0.000

For Pearson's correlation (Table 6), none of the pair was observed with a negative correlation however, the drawback of “mentioning the baseline characteristics for the groups” was found to have no significant correlation with any other drawback extracted from the study. With the same concept of evidence, PCA showed no loading with significant Eigen value for this drawback. The reason is due to sparse distribution of current drawback in the reported clinical trials. The Pearson's correlation between “intention-to-treat-analysis” and “outcome assessment method” again confirms the loading for both the drawbacks in PC3, and so on. The descending order of the drawbacks in these clinical trials, based on PCA and Pearson's correlation may be constructed as;

**Table 6:** Pearson correlation matrix for factors observed in clinical trials (letter A-L represents the points as mentioned in Table 3).

	**A**	**B**	**C**	**D**	**E**	**F**	**G**	**H**	**I**	**J**	**K**	**L**
**A**	**1**											
**B**	**0.394**^∗^	**1**										
**C**	**0.305**^∗^	**0.459**^∗^	**1**									
**D**	**0.285**^†^	−0.052	0.181	**1**								
**E**	**0.389**^∗^	**0.771**^∗^	**0.699**^∗^	0.067	**1**							
**F**	0.157	0.176	**0.656**^∗^	0.207	**0.600**^∗^	**1**						
**G**	**0.252**^†^	0.223	**0.242**^†^	0.108	**0.248**^†^	−0.034	**1**					
**H**	**0.423**^∗^	**0.252**^†^	**0.383**^∗^	0.184	**0.372**^∗^	**0.251**^†^	**0.330**^∗^	**1**				
**I**	0.222	−0.076	0.105	0.063	0.043	0.155	0.149	**0.343**^∗^	**1**			
**J**	−0.062	0.115	0.115	−0.144	0.029	−0.106	**0.270**^†^	0.138	0.039	**1**		
**K**	0.108	0.140	0.095	0.037	0.154	0.052	0.052	0.099	0.180	0.070	**1**	
**L**	0.087	−0.151	0.012	0.081	0.077	**0.261**^†^	0.013	0.154	**0.278**^∗^	−0.051	0.080	**1**

[”Non-randomized and non-blinded,” “concealed treatment allocation,” “non-blindness of patient and care provider,” “lack of drop out procedures”] > [“lack of patients compliance and co-interventions reports”] > [“non-inclusion of intention-to-treat-analysis and timing of outcome assessment”] > [“outcome assessor non-blind”] > [missing to mention the baseline characteristics of the groups].

**Figure 4:**
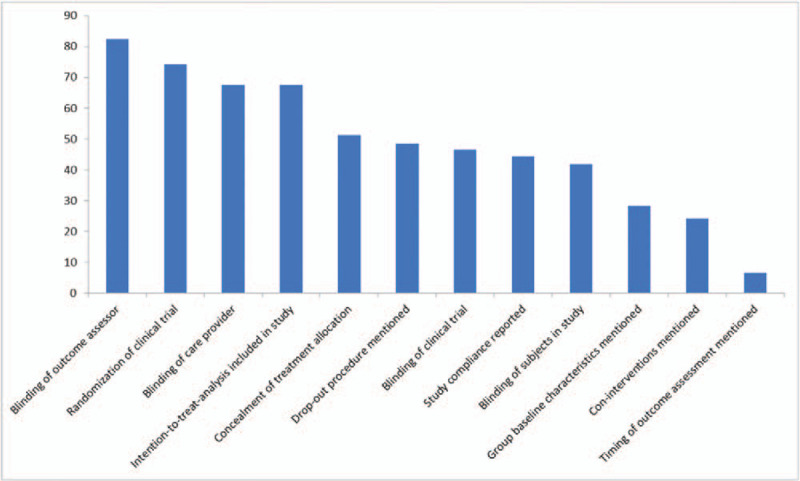
Descending order for drawbacks based upon “no” or “not reported” responses extracted from clinical.

Figure 4 represents the descending order of occurrence for these drawbacks.

## 4 Scoring of clinical trials (jadad, Delphi, and, cochrane scales)

For simplicity and leniency, an internal grading scale (points) was applied; 6 or below (any negative value) “very poor quality clinical trial,” 7 to 12 “poor quality clinical trial,” 13 to 18 “acceptable quality clinical trial,” and 19 to 24 “good quality clinical trial.” Beside the two excellent studies (D10, I8) the percentage for good quality clinical trial observed was 13.5%. The very poor and poor quality clinical trials makes the major proportion (67.6%) of these studies (Table 7).

**Table 7:** Scoring for clinical trials based on in-house grading scale (0–24 points).

Quality of trial based on assigned scale	Frequency (N)	Percent (%)	Cumulative Percent
Very poor quality clinical trials (6 and below, i.e., negative value)	25	33.8	33.8
Poor quality clinical trials^[7–12]^	25	33.8	67.6
Acceptable quality clinical trials^[13–18]^	14	18.9	86.5
Good quality clinical trials^[19–24]^	10	13.5	100.0
Total	74	100.0	

## 5 Discussion

This study presents a generalized view of the major drawbacks observed in the clinical trials selected and evaluated. The forthcoming discussion is a section-wise classification with extensive explanation of the drawbacks extracted;

### 5.1 Selection and identification of the plant source

To commence a clinical trial, the identification and quality of the source (natural product) is utmost important. Sufficient background information regarding the identity of the selected source is very essential. Plants may vary in terms of quality and quantity of the active principles present which is due to variation in geographical origins. Hence, the differences in environment, temperature, irrigation, salinity, stress, altitude, and seasonal variation may affect the composition of plant phytochemicals. A lack of very basic and essential information about the herbs used (family, genus, species and geographical origin, phytochemistry and quantification, and mechanism of actions, etc) was witnessed in these studies, which are strictly needed for a herb to be worked on as mentioned by Heinrich et al.^[20]^ Such information regarding the species and its phytochemistry may help researchers and stakeholders to dig deeper at mechanistic and molecular level to isolate the compound of interest for an effective control of diabetes and related comorbidities. The proper evidence regarding ethnopharmacological and ethnomedicinal value of the plants reported,^[21,22]^ is of prime importance.

### 5.2 Part of natural product used and its phytochemistry

Every individual part of a plant may vary with respect to the other part of the same plant in terms of nature of active ingredients or even the amount of similar active ingredient.^[23]^ For instance, the antidiabetic effect was studied for seeds and leaves of Jambolan in these clinical trials. The seeds were observed with prominent antidiabetic effect whereas, the leaves for the same plant exhibited a lack of activity in diabetes. This is a self-explanatory evidence to choose wisely during selection of plant part as it may vary in phytochemistry.

### 5.3 Effects of extraction methods, temperatures and solvents upon the final dosage form preparation

The process-selection for dosage form preparation (extract, tea, infusion, essential oil, etc), exerts a subtle difference in the final outcomes of a study. The phenomenon has been mentioned in very detail in previous reports.^[24–26]^ For example, Nopal was applied in the form of steamed as well as broiled steamed dosage form in these reported clinical trials. A proper explanation is missing regarding part, dosage form of Nopal used, and the effect of heat/difference in composition of steamed and broiled steamed Nopal dosage form in terms of active drug amount and therapeutic effects.

### 5.4 Use of toxic and hazardous solvent for dosage form preparation

The use of toxic and hazardous solvents for extraction may be problematic. One of the clinical trial for fenugreek applied hydro-alcoholic solvent for extract preparation. Another clinical study for bitter melon applied a technique for proper phytochemical screening which is interesting to see however, the same study utilized toxic solvents of carbon tetra chloride and benzene for extraction which are well known to release highly toxic phosgene fumes when heated. Hence, toxicity profile needs to be reported for these extracts in preclinical studies. An effective widely used alternative approach now-a-days is, the use of green solvent and green extraction.^[27]^

### 5.5 Clinical phase selection for study (Phase 0-V)

Phase-0 is a preliminary step in any clinical study where sub-therapeutic dosing is tested in about ten individuals in order to determine the pharmacokinetics and pharmacodynamics profile.^[28]^ This supportive data helps to safely administer any herb or herbal product in human participants, without compromising the subject's rights and values. The data regarding toxicity and sub-therapeutic dosing, that is, Phase-0, is not included in any clinical trial mentioned here. In addition, the clinical trials in this systemic review are mostly reported at a preliminary level of Phase-I/II which necessitates a major data regarding Phase III–V.

### 5.6 Blinding and concealment of study

Natural products such as cinnamon, onion, and garlic possesses a particular smell/aroma and taste, the masking of which is utmost important to avoid any ambiguities/biasness in a study. Furthermore, to avoid any data manipulation, clinical studies needs to be of double-blind nature where both; the care provider/product administrator and outcome assessor are blinded towards the product used, protocols applied, subjects group assigned, and parameters/data to be studied.^[29,30]^ Unfortunately, majority of the clinical trials herein were observed to be non-blinded or single blinded on behalf of patients, health care providers or outcome assessors.

### 5.7 Duration and compliance of clinical trials

The duration for these clinical trials ranged from week to months including 6 (cinnamon) and 20 months (gymnema) which are too lengthy and may be pose risks in certain conditions. No doubt, diabetic patients are asked to continue antidiabetic medications on a regular basis however, the co-administration of a natural product with conventional medication may produce unavoidable circumstance due to potential herb-drug interactions in such chronic used patient. Thus more lengthy studies with frequent dosing may lead to non-compliance which was observed in all of the clinical trials reported here.^[31–33]^

### 5.8 Potential herb–drug interactions

Severe or life threatening adverse effects have been reported when herbs are co-administered with conventional medicines. For example, bitter melon alongwith oral hypoglycemic may decrease blood glucose level^[34,35]^ whereas, *Aloe vera* alongwith insulin/oral hypoglycemic potentiates the hypoglycemic effect hence, needs a proper caution.^[34,36]^ These clinical trials were unable to explain; how to co-administer the plant with conventional drugs? What is the half-life, excretion ratio/rate, plasma protein binding, and clearance mechanism for extract/herbs studied? Most of the clinical trials used Metformin (half-life: 6.2 h); however, none of them mentioned the dosage frequency for the natural product, information about co-administration, dosage-gap, half-life of natural products used, metabolic pathways and enzymes involved, as well as any herb-drug interactions for the products studied. For example, clinical trial for turmeric and metformin observed a pronounced effect, yet no any prior information were available for such effect.

### 5.9 Dose of the natural product used

A dose of 1 to 6 g/day was observed in most of these clinical trials which apparently seems too high for any health related condition treatment. The question rises of how the powder/extract was converted to tablet/capsule with such a high amount? Based on size availability, “000” capsule is able to encompass a dose up to 1 g (subjected to natural product density as it may can reduce to 700/800 mg in case of dense powders). Above all, administering the allowable powder amount/capsule with a frequency of three time/day is still unable to deliver a dose of 3 g or above whereas, a number of clinical trials reported a dose up to 6 g/day. In particular, patient using conventional drugs (on conventional therapy) were given such high doses 3 to 4 times/day. This is a high risk trial with more chances and risks of life threatening herb-drug interactions. These studies completely skipped/failed to report proper information for managing the high doses/day in capsules, especially cinnamon (6 g), fenugreek (10 g), and nopal (500 g).

### 5.10 Placebo or psychological effect

Improper masking in some cases may produce psychological effects. One of the reported study using coconut oil for a placebo group observed alike effects in the all groups^[37]^ which was explained due to a psychological phenomenon related to placebo. Most of the clinical trials reported herein were observed with a lack of proper coding/process, for natural products carrying intense smell.

## 6 Future perspectives and recommendations

### 6.1 Ethnopharmacological relevance of the plant with the study

To conduct a herbal clinical trial, it is essential to know its source (family, genus, species), ethnobotanical/ethnopharmacological relevance with the study, part of the plant used and phytochemical profile, preclinical, and toxicity data available as well as the information regarding the PKs and PDs of the herbal active ingredients.^[20–22]^

### 6.2 Identification and geographical information

Geographical location may affect the quality and quantity of active ingredient in a plant thus proper identification of geographical locations alongwith preliminary quality variation and standardization studies play a vital role for unification of the source intended for any clinical trial.^[38,39]^ The pharmacological activities and toxicity profile must be ensured in animal and in vitro models as this will ease the uniformity of dose applied for an optimum activity of the plant.

### 6.3 A need for extraction or isolation does exist?

Albeit, it's a tiresome job with much investment to isolate an active chemical whereas, herbal extract/powders are easy to apply/use which adds an additional advantage of synergism. This made a dogma shift more toward the use of herbs and herbal products. However, when it comes to composition, quantity, mechanism of action, and PKs and PDs of the active drug, its tricky at times to study herbal samples as they are mixture of multicomponent.^[40]^ Thus isolation of active principle is highly recommended as it is far better in order to evaluate the effect of a herbal drug at molecular or genetic level and to market the lead molecule as a potential drug candidate. For instance, the hypoglycemic polypeptide-p or p-insulin in *Momordica charantia*^[41]^ is yet to be marketed. Likewise, eleutherosides presence makes Siberian ginseng more effective compared to *Panax ginseng* as a proper mechanism has been proposed for eleutherosides.^[42]^

### 6.4 Selection and preparation of final dosage

Due to variation in extraction tools (shorter and greener extraction) and parameters (temperature, solvent, particle size, length of extraction, pressure used during extraction and combination of solvents), the final dosage form may vary in concentration and activity of the active drug.^[24–26]^ Powder samples (more surface area) are known to enhance the solubility, absorption, PKs, and PDs of the herbal drugs or extracts. For example, the clinical trial for psyllium observed a marked difference in activity for soluble fibers Vs whole seeds. Similarly, bitter melon juice showed pronounced effect compared to the fruit powder. For temperature effect, dehydrated Nopal study showed no results whereas, for drying effect, shade dried bitter melon revealed no effect compared to fresh and unripe fruit. Currently, the researchers are focusing to prepare the nano-dosage forms (nano-gels, micelles, nanoparticles and nanoemulsions) which are more target-oriented and effective at low doses. Curcumin-nano-micelle in one clinical trial exhibited excellent results.

### 6.5 Dataset available for clinical phases studied (Phase 0-V)

Proper literature data for Phase 0-V is necessary. Phase-0, though necessary to determine the sub therapeutic/starting dose/toxicity and PKs in human, is often not mandatory for plants with well-known ethnopharmacological background. Thus background information is very important to decide the clinical trials phase.^[43]^

### 6.6 Implementation of plan for short duration, multicenter and large scale study

Clinical trials with shorter duration are more effective, particularly in a population where the patient is either asked to pause/drop the conventional therapy during a clinical study or the subjects are maintained on conventional and natural products together. This will avoid unnecessary life threatening consequences due to herb–drug interactions.^[29,30]^

### 6.7 Strategy for treating co-markers or stress-related-markers in diabetes

A high level of stress prevalence have been reported in diabetic population. Various byproduct such as Amadori, advanced glycation end products (glyoxal, methyl glyoxal, fructosamine, etc), or inflammatory (interleukin-1β, insulin-like growth factor 1 [IGF-1] and monocyte chemoattractant protein 1, etc) and stress-related markers (thiobarbituric acid-reactive substances, glutathione, Catalase, Superoxide dismutase, etc) may be interesting and fruitful to study. These clinical trials may help eliminate/reduce the burden of diabetes and its comorbidities.^[44,45]^

### 6.8 Translation of the clinical trials into special populations

In addition to adult male/female subjects, it would be more helpful to conduct these clinical trials with caution in special population (pregnant, breast feeding women, and geriatric).

### 6.9 The need for a mechanistic approach

The herbs/herbal mixture may be challenging to determine the phytochemical profile hence, isolation is more preferable to overcome this loophole. However, if not required, a proper phytochemistry, suggested mechanism of action, metabolizing enzymes, and clearance pathways are least to be determined for any herbal product to be studied in a clinical trial.

## 7 Conclusion

Apart from the loopholes and drawbacks of the clinical studies reported, the authors worked to add few additional important points (Table 8), which includes; the availability of data regarding ethnopharmacological relevance, pharmacovigilance of medicinal plant (identification of plant and its part to be used with proper phytochemical profile) and intention to focus on co-markers in diabetes. These points may facilitate researchers to plan a clinical trial with high quality and uniformity.

**Table 8:** Additional points suggested by authors for quality of clinical trials and its evaluation.

Ethnopharmacological relevance of the plant	Proper preliminary literature or data (in vitro animal models, cell culture studies, etc) needed to establish the role in diabetes
Identification and taxonomy of planta. information regarding taxonomy	Identification of the correct plant and its part via authentic sources including herbarium, taxonomist, botanist and botanical gardens
b. information about plant part to use	Phytochemical screening for different plant parts with in vivo and in vitro models of pharmacological activities
c. quality variation and standardization	Quality variation for the same plant in terms of different geographical origins need to be standardized through green, short and reproducible analytical and pharmacological tools
Pharmacovigilance data of plant	Safety and efficacy data for Phase-0 with an extensive preclinical model for toxicity studies needed in order to rule out the side and adverse effects at a proper dose
Inclusion of co-markers treatment	Various non-conventional markers of stress, inflammation, glycation end products, etc, needs to be evaluated alongside the conventional markers

## 8 Final note

The aim of the study was purely to evaluate the quality of reported clinical trials for natural products in diabetes and it does not aim to establish superiority of one clinical trial over the other nor does it intend to provide a comparison for the quality of researchers.

## Author contributions

RA conceived the idea and designed the study with LHA and HNA. RA and all authors conducted literature review and wrote the introduction. RA, LHA, HNA along with MAA wrote the methodology. All the authors contributed in evaluation of each and individual clinical trial and scoring them on an excel sheet. LHA and HNA alongwith AFA, LSA, KNA, and HJA wrote the body parts including; ethnopharmacological relevance, clinical trials evaluation and schedules. RA wrote the discussion and future prospective whereas RA along with LHA and MAA performed statistical analysis. RA edited the final draft and all the authors reviewed and approved the final manuscript.

**Conceptualization:** Rizwan - Ahmad, Lina Hussain AlLehaibi.

**Data curation:** Rizwan - Ahmad, Lina Hussain AlLehaibi, Hind Nasser AlSuwaidan.

**Formal analysis:** Rizwan - Ahmad.

**Funding acquisition:** Majed A Alkhathami.

**Investigation:** Rizwan - Ahmad.

**Methodology:** Rizwan - Ahmad, Ali Fuad Alghiryafi, Lyla Shafiq AlMubarak, Khawlah Nezar AlKhalifah, Hawra Jassim AlMubarak.

**Resources:** Hind Nasser AlSuwaidan, Ali Fuad Alghiryafi, Khawlah Nezar AlKhalifah, Majed A Alkhathami.

**Software:** Hawra Jassim AlMubarak.

**Supervision:** Rizwan - Ahmad.

**Validation:** Rizwan - Ahmad.

**Writing – original draft:** Rizwan - Ahmad, Lina Hussain AlLehaibi.

**Writing – review & editing:** Rizwan - Ahmad, Hind Nasser AlSuwaidan, Ali Fuad Alghiryafi, Lyla Shafiq AlMubarak, Khawlah Nezar AlKhalifah, Hawra Jassim AlMubarak, Majed A Alkhathami.
